# The obesity paradox and 90 day mortality in chronic critically ill patients: a cohort study using a large clinical database

**DOI:** 10.1186/s40001-024-01962-w

**Published:** 2024-07-29

**Authors:** Danyu Xu, Yan Lu, Yan Wang, Feng Li

**Affiliations:** 1 Chongqing Medical University, Chongqing, 400016 People’s Republic of China; 2 The First Affiliated Hospital of Chongqing Medical University, Chongqing, 400016 People’s Republic of China

**Keywords:** CCI, MIMIC-IV database, Body mass index, Mortality, Obesity paradox

## Abstract

**Background:**

This study investigates the obesity paradox, where obesity is linked to lower mortality in certain patient groups, focusing on its impact on long-term mortality in chronic critically ill (CCI) patients.

**Methods:**

We retrospectively analyzed CCI patients from the Medical Information Mart for Intensive Care-IV (MIMIC-IV) database’s Intensive Care Unit, categorizing them into six groups based on Body Mass Index (BMI). Using stepwise multivariable Cox regression and restricted cubic spline models, we examined the association between BMI and 90 day mortality, accounting for confounding variables through subgroup analyses.

**Results:**

The study included 1996 CCI patients, revealing a 90 day mortality of 34.12%. Overweight and obese patients exhibited significantly lower mortality compared to normal-weight individuals. Adjusted analysis showed lower mortality risks in overweight and obese groups (HRs 0.60 to 0.72, *p* < 0.001). The cubic spline model indicated a negative correlation between BMI and 90 day mortality, with subgroup analyses highlighting interactions with age.

**Conclusion:**

Our findings confirm the obesity paradox in CCI patients, especially among the elderly (65–85 years) and very elderly (≥ 85 years). The results suggest a beneficial association of higher BMI in older CCI patients, though caution is advised for those under 45.

**Supplementary Information:**

The online version contains supplementary material available at 10.1186/s40001-024-01962-w.

## Background

The advancement of intensive care medicine has improved the prognosis of critically ill patients. However, some patients who experience acute critical illnesses retain persistent organ dysfunction and require long-term intensive care unit management, turning into chronic critical illness (CCI) [1]. Several studies have shown that the incidence of CCI exceeds 30% [2–4], and the incidence of CCI in COVID-19 patients is even higher [5]. The 1 year mortality of CCI patients can reach as high as 50–60% [6], requiring more medical resources with lower survival quality, high readmission rate, and poor long-term prognosis, thereby increasing the burden on families and society [7]. The risk factors for CCI include age, comorbidities (such as hypertension and diabetes), sepsis, surgical intervention, and multiple organ failure [8–11]. It is also found that overweight and obesity were possible risk factors for CCI [12]. However, only a few studies have been conducted on the risk factors for patient prognosis in CCI cases.

The prevalence of obesity has seen a significant rise due to changes in lifestyle and dietary habits. According to data 2016, it is estimated that by 2025, the global male obesity rate will reach 18%, and the female obesity rate will exceed 21% [13]. Obesity has multiple effects on the body, and in patients, obesity is generally associated with an increased risk of developing chronic conditions such as hypertension and diabetes [14], which results in higher morbidity and mortality compared to non-obese patients. Additionally, obese patients have demonstrated significantly higher re-admission rates and mortality than non-obese patients [15]. For critically ill patients, approximately three-fourths of ICU patients are either overweight or obese [16]. Obesity greatly increases the risk of respiratory complications such as difficult airways and atelectasis, especially in critically ill patients [17]. Moreover, conventional fluid resuscitation strategies may not be applicable to septic obese patients [18]. In addition, obesity is an independent risk factor for acute kidney injury (AKI) in critically ill patients [19]. Therefore, special attention should be given to obese, critically ill patients. 

Although obesity has obvious disadvantages for patients, researchers have observed the obesity paradox in recent years. however, it paradoxically improves patients’ prognosis. Various studies have confirmed the existence of this phenomenon in critical illnesses [20–23], including COVID-19 [24]. Recent studies have found that this phenomenon also exists in the long-term prognosis of critically ill patients [25, 26]. However, while many researchers support this paradox, others hold the opposite opinion. For instance, it has been reported that obesity did not improve the outcomes in COVID-19 patients requiring invasive mechanical ventilation [27]. Additionally, high BMI was not associated with survival or time on ECMO [28]. Some studies also suggest that BMI is not related to the prognosis of critically ill patients [29–31]. Nevertheless, whether the obesity paradox exists in CCI patients has not been reported before.

The present study used the Medical Information Mart for Intensive Care-IV (MIMIC-IV) database, the most widely used open database in the field of critical care medicine [32], to investigate whether the obesity paradox exists in CCI patients and whether BMI has an impact on their 90-day mortality.

## Methods

### Data sources

All related data were extracted from the MIMIC (MIMIC-IV 2.0 version) database, updated on June 22nd, 2022, and death records were filled in after discharge, allowing for a better study of the long-term prognosis of critically ill patients. Researchers accessed and extracted the data after obtaining the required training courses and certification from the National Institutes of Health (Certification No.: 36743986).

### Study population and criteria

Patients from the MIMIC-IV database who met the following criteria were selected for this study. Firstly, patients who met the definition of CCI [2, 3] with more than 14 days in ICU and persistent organ dysfunction (Sequential Organ Failure Assessment (SOFA score) ≥ 2) at day 14. Secondly, participants had to be at least 18 years old. Thirdly, patients must have available body mass index (BMI) data, which was calculated as weight (in kilograms) divided by height (in meters) squared. For patients admitted multiple times, only the first ICU admission information was included in this study. Patients who failed to meet any of these criteria were excluded from this study.

### Outcomes

The primary endpoint of this study was 90 day mortality, while the secondary endpoints included ICU length of stay, hospital length of stay, ICU mortality, hospital mortality, 28 day mortality, and 1 year mortality.

### Data extraction

All data in this study were obtained through Structured Query Language (SQL), including general demographics (age, gender), laboratory parameters (renal function, coagulation function, electrolytes, blood gas, etc.), comorbidities (hypertension, diabetes, chronic heart failure, and Charlson comorbidity index), organ support [vasoactive agents, invasive mechanical ventilation, and continuous renal replacement therapy (CRRT)], severity score (Simplified Acute Physiology Score II (SAPS II) and SOFA), and clinical outcomes (ICU mortality, hospital mortality, 28 day mortality, 90 day mortality, 1 year mortality). Laboratory parameters with > 5% missing values were excluded from this study. For parameters with missing values ≤ 5%, values were imputed using median (for non-normally distributed data) or mean (for normally distributed data).

### Statistical analysis

All data of this study were analyzed using the STATA 16.0 software. All patients were categorized into six groups based on the World Health Organization BMI (WHO BMI) classification: underweight group (BMI < 18.5 kg/m^2), normal group (BMI 18.5–25 kg/m^2), overweight group (BMI 25–30 kg/m^2), obesity class I group (BMI 30–35 kg/m^2), obesity class II group (BMI 35–40 kg/m^2) and obesity class III group (BMI > 40 kg/m^2). Continuous variables were expressed as mean ± standard deviation (SD) or median (interquartile range), and single factor variance analysis (ANOVA) or Kruskal–Wallis test was used according to whether the distribution was normal. Categorical variables were expressed as percentages and tested using χ^2^tests.

Kaplan–Meier curves were plotted for each group to analyze 90 day mortality, and a log-rank test was used to identify the differences between groups. Univariate and multivariate Cox proportional hazards regression models were conducted to calculate the adjusted risk ratios (HRs) and 95% confidence intervals (95% CI) to evaluate the impact of each predictor on 90 day mortality of CCI patients. The univariable Cox proportional hazards regression model was used to identify prognostic variables. Stepwise Cox regression was used to identify the independent influence variables. In the multivariable analysis, various statistical models were applied to ensure the reliability of the results. Model I had no adjustments in any of the variables. Model II was adjusted for age and gender. Model III was further adjusted for potential confounders that were identified in the stepwise Cox regression with a *p* value of < 0.05. The 18.5–25 kg/m^2 group was used as the reference standard for the BMI categorical variables.

The restricted cubic spline models fitted with three knots at the 25th, 50th, and 70th percentiles of BMI were used for multivariate Cox proportional hazards regression model III to investigate the association between BMI as a continuous variable and 90 day mortality in CCI patients. To exclude the influence of confounders, subgroup analysis was performed to compare the influence of BMI on the 90 day mortality of CCI patients. All probability values were 2-sided, and values of less than 0.05 were considered statistically significant.

## Results

### Patient characteristics

**Fig. 1:**
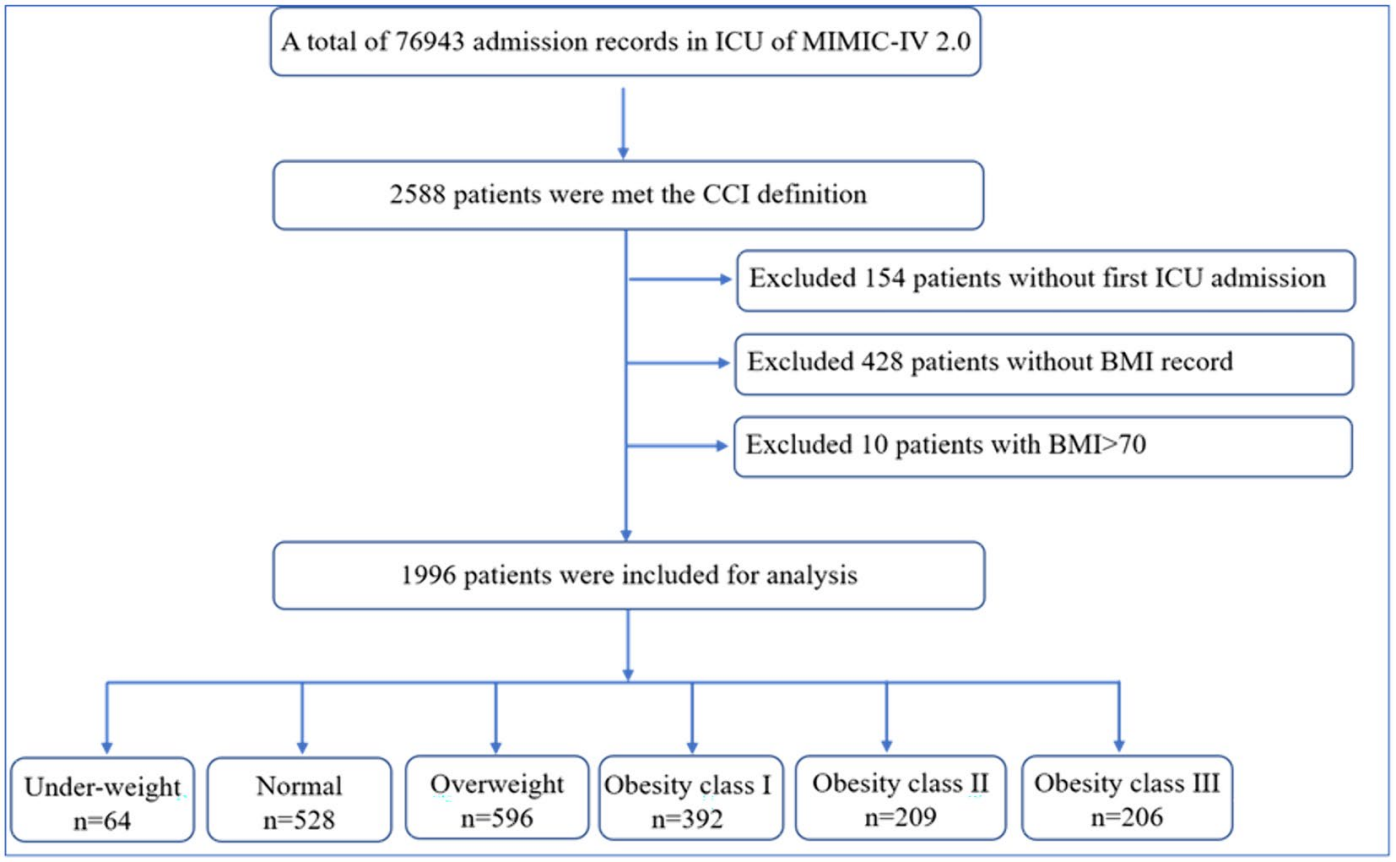
The study flowchart

After the final screening, a total of 1996 eligible patients were included in the analysis, as shown in Fig. 1. All patients were divided into six groups, among which the underweight group had 64 patients, accounting for 3.21% of CCI patients; the normal group had 528 patients, accounting for 26.45% of all CCI patients; the overweight group had 597 patients, accounting for 29.91% of all CCI patients; and the obesity group had 807 patients, accounting for 40.43% of all CCI patients, among which obesity class I group accounted for 19.64% patients, obesity class II group accounted for 10.47% patients and obesity class III group accounted for 10.32% patients.

The general characteristics, laboratory parameters, blood gas, vital signs, comorbidities, disease severity scores, and prognosis of the four groups are shown in Table 1. The results showed that the groups with higher BMI were of a younger age than the underweight and normal groups. Additionally, a male predominance was observed in the overweight and obesity class I groups. Regarding laboratory parameters, the overweight and obesity groups had lower red cell distribution width (RDW), higher creatinine and urea nitrogen (BUN), longer prothrombin time (PT) and international normalized ratio (INR), and lower arterial partial pressure of oxygen (PaO2) compared with underweight and normal groups. The groups with higher BMI were more likely to be associated with a higher incidence of hypertension and diabetes, a higher SOFA score, and a higher proportion of CRRT during hospitalization.

**Table 1:** Characteristics of the patients according to BMI categories

Variables	Under-weight group (n = 64, 3.21%)	Normal group (n = 528, 26.45%)	Overweight group (n = 597, 29.91%)	Obesity class I group (n = 392, 19.64%)	Obesity class II group (n = 209, 10.47%)	Obesity class III group (n = 206, 10.32%)	*p* value
Demographic characteristics							
Age (years)	65.31 ± 18.13	64.92 ± 17.18	63.77 ± 15.97	62.65 ± 14.70	63.42 ± 14.09	59.78 ± 13.48	< 0.001
Male n (%)	34 (53.12)	300 (56.81)	383 (64.15)	259 (66.07)	109 (52.15)	99 (48.05)	< 0.001
White	34 (53.13)	319 (60.42)	390 (65.33)	257 (65.56)	122 (58.37)	128 (62.14)	0.130
Admission type							0.000
Emergency	34 (53.12)	290 (54.92)	320 (53.60)	199 (50.77)	98 (46.89)	107 (51.94)	
Urgent	9 (14.06)	89 (16.86)	131 (21.94)	102 (26.02)	69 (33.01)	64 (31.07)	
Surgery	15 (23.44)	111 (21.02)	111 (18.59)	69 (18.55)	32 (15.31)	23 (11.17)	
Selected	6 (9.38)	38 (7.20)	35 (5.86)	22 (5.61)	10 (4.78)	12 (5.83)	
Laboratory parameters							
Hemoglobin (g/dl)	10.49 ± 2.73	10.79 ± 2.47	11.02 ± 2.62	11.16 ± 2.77	10.91 ± 2.63	11.52 ± 2.64	0.255
WBC (× 10^9^/L)	11.25 (9.00–16.55)	11.3 (7.9–15.7)	12.3 (8.1–17.1)	12.10 (8.50–17.40)	12.70 (9.10–17.80)	13.25 (8.80–17.40)	0.076
PLT (× 10^9^/L)	219.50 (146.00–333.50)	195.00 (131.00–272.00)	202.00 (138.00–274.00)	190.00 (125.00–247.00)	199.00(144.00–269.00)	214.50(145.00–286.00)	0.017
RDW (%)	15.75 ± 2.59	15.06 ± 2.41	15.41 ± 2.71	15.24 ± 2.48	15.65 ± 2.44	15.80 ± 2.56	< 0.001
Creatinine (mg/dl)	0.90 (0.65–1.50)	0.90 (0.70–1.40)	1.10(0.80–1.60)	1.30 (0.90–2.10)	1.40 (0.90–2.30)	1.30 (0.90–2.10)	< 0.001
BUN (mg/dL)	21.50 (13.00–31.50)	19.00 (13.00–31.00)	21.00(14.00–35.00)	23.00 (15.00–41)	26.00 (16.00–48.00)	25.00 (17.00–42.00)	< 0.001
PT (S)	13.45 (11.90–15.50)	13.70(12.20–16.70)	14.00(12.50–18.20)	14.35 (12.60–18.60)	14.20 (12.60–17.60)	14.50 (12.60–17.80)	0.008
APTT (S)	31.85(28.55–40.10)	31.15 (27.30–39.00)	31.30 (27.00–40.80)	31.80 (26.90–42.30)	30.70 (26.70–37.00)	30.40 (26.80–38.30)	0.359
INR	1.20 (1.10–1.40)	1.25(1.10–1.50)	1.30 (1.10–1.70)	1.30 (1.10–1.70)	1.30 (1.10–1.60)	1.30 (1.20–1.60)	0.012
Potassium (mmol/L)	4.41 ± 1.03	4.16 ± 0.85	4.27 ± 0.81	4.38 ± 0.90	4.44 ± 0.91	4.57 ± 0.98	0.004
Sodium (mmol/L)	140.50 ± 7.68	138.34 ± 6.11	138.18 ± 6.07	138.20 ± 5.63	138.39 ± 5.69	138.09 ± 5.61	0.009
Chloride (mmol/L)	104.96 ± 8.22	103.37 ± 7.75	102.94 ± 7.58	102.62 ± 7.11	102.62 ± 7.02	101.86 ± 7.04	0.018
Calcium (mg/dL)	8.37 ± 0.87	8.3 ± 1.03	8.32 ± 1.06	8.31 ± 1.06	8.28 ± 1.05	8.34 ± 1.10	0.362
Glucose (mg/dL)	116.00 (103.00–150.50)	133.00(108.00–172.00)	132.00(109.00–173.00)	148.00(113.00–191.00)	142.00 (112.00–182.00)	148.00 (116.00–194.00)	< 0.001
Blood gas							
pH	7.34 (7.27–7.43)	7.38 (7.30–7.43)	7.38 (7.28–7.44)	7.36 (7.28–7.42)	7.35 (7.27–7.42)	7.34 (7.27–7.40)	0.001
PaO_2_ (mmHg)	105.00 (61.00–182.00)	125.00 (68.00–242.00)	110.00 (68.00–193.00)	104.00 (70.00–184.00)	91.00 (65.00–165.00)	89.00 (62.00–125.00)	< 0.001
PaCO_2_ (mmHg)	41.00 (36.00–58.00)	40.00 (34.00–46.00)	39.00 (34.00–47.00)	41.00 (35.00–48.00)	43.00 (36.00–53.00)	43.00 (37.00–52.00)	< 0.001
Anion gap (mmol/L)	15.00 (12.00–18.00)	15.00 (13.00–18.00)	16.00 (13.00–19.00)	16.00 (13.00–20.00)	15.00 (13.00–18.00)	15.00 (13.00–19.00)	0.005
Bicarbonate (mmol/L)	22.00 (19.50–26.00)	22.00 (19.00–25.00)	22.00 (19.00–25.00)	22.00 (18.00–25.00)	22.00 (19.00–26.00	23.00 19.00–27.00)	0.118
Lactate (mmol/L)	1.60 (1.10–2.50)	1.70 (1.10–2.80)	1.80 (1.20–3.00)	1.90 (1.30–3.20)	1.80 (1.20–3.10)	1.80 (1.25–2.85)	0.041
vital signs							
Heart rate (beats/minute)	92.32 ± 21.00	92.71 ± 21.27	91.95 ± 22.11	93.47 ± 21.68	92.70 ± 23.46	94.92 ± 20.69	0.482
Respiratory rate (beats/minute)	20.79 ± 7.02	20.64 ± 6.59	20.92 ± 6.80	21.12 ± 6.28	21.22 ± 7.02	21.83 ± 5.96	0.107
Temperature (°C)	36.42 ± 1.09	36.69 ± 0.92	36.69 ± 1.10	36.78 ± 1.09	36.79 ± 1.19	36.90 ± 0.82	< 0.001
MBP (mmHg)	83.50 (69.50–97.00)	82.00 (71.00–97.00)	82.00 (70.00–95.00)	82.00 (71.00–96.00)	81.00 69.00–95.00)	81.00 (68.00–94.00)	0.750
SPO_2_ (%)	99.00 (96.00–100.00)	99.00 (96.00–100.00)	98.00 (95.00–100.00)	98.00 (94.00–100.00)	98.00 (94.00–100.00)	96.00 (93.00–99.00)	< 0.001
Comorbidities, n (%)							
COPD	8(12.50)	39(7.39)	29(4.86)	31 (7.91)	21 (10.05)	26 (12.62)	0.004
Congestive heart failure	2 (3.13)	31 (5.87)	39 (6.53)	28 (7.14)	17 (8.13)	17 (8.25)	0.621
Diabetes	8 (12.50)	108(20.45)	165(27.64)	155 (39.54)	92 (44.01)	100 (48.54)	< 0.001
Hypertension	17(26.56)	194(36.74)	210 (35.17)	169 (43.11)	83 (39.71)	82 (39.23)	0.020
Charlson comorbidity index	6.00 (5.00–8.00)	6.00 (4.00–8.00)	6.00 (5.00–8.00)	6.00 (4.00–8.00)	6.00 (4.00–8.00)	5.00 4.00–7.00)	0.254
Severity Scoring systems							
SAPS II	41.00 (35.00–56.00)	42.00 (32.50–53.50)	42.00 (32.00–53.00)	44.00 (34.00–53.00)	45.00 36.00–55.00)	42.00 (33.00–50.00)	0.092
SOFA	8.00 (5.00–11.50)	8.00 (5.00–11.50)	9.00 (6.00–12.00)	10.00 (7.00–13.00)	10.00 (7.00–13.00)	9.00 (7.00–12.00)	< 0.001
Organ support, n (%)							
CRRT	9 (14.06)	87 (16.47)	156 (26.13)	116 (29.59)	70 (33.49)	62 (30.09)	< 0.001
Invasive ventilation	62 (96.88)	512 (96.97)	569 (95.31)	376 (95.92)	201 (96.17)	196 (95.15)	0.766
Vasoactive agent	49 (76.56)	431 (81.63)	477 (79.90)	324 (82.65)	171 (81.82)	165 (80.09)	0.803
Sepsis n (%)	28 (40.63)	196 (37.12)	225 (37.69)	159 (40.56)	81 (38.76)	102 (49.51)	0.044

Vasoactive agent included noradrenaline, vasopressin, phenylephrine, epinephrine, dopamine

*S* seconds, *MBP* Mean blood pressure, *pH* power of hydrogen, *PaO*_*2*_ Arterial partial pressure of oxygen, *PaCO*_*2*_ Arterial partial pressure of carbon dioxide, *SPO*_*2*_ Peripheral capillary oxygen saturation, *COPD* Chronic obstructive pulmonary disease

### Composition of patients with CCI

**Fig. 2:**
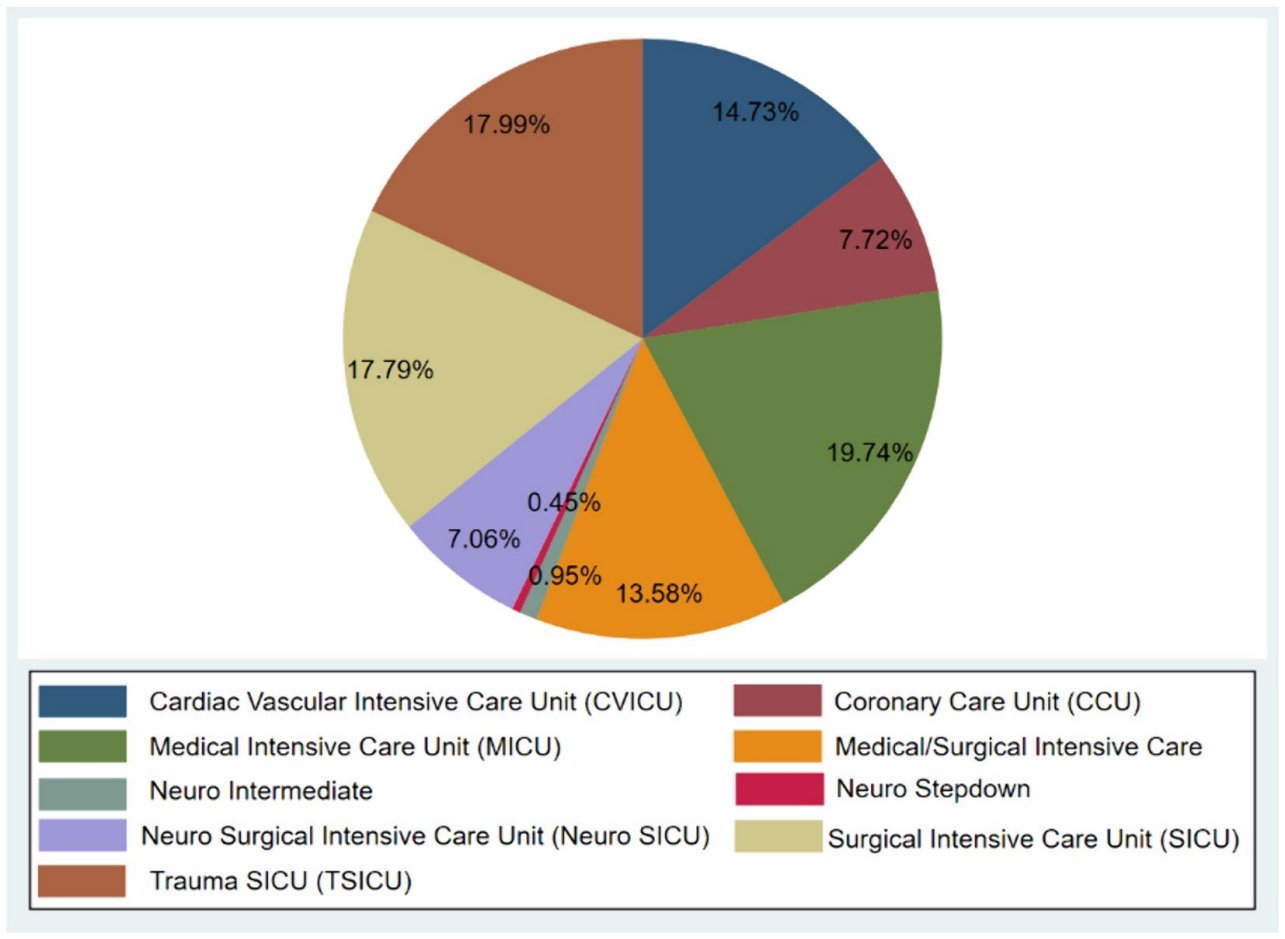
The composition of patients with CCI

To further investigate the clinical characteristics of CCI patients, the distribution of patients was analyzed. The predominant patients in the ICU were from the Medical Intensive Care Unit (MICU) (19.74%), followed by the Trauma Surgical Intensive Care Unit (TSICU) (17.99%) and the Surgical Intensive Care Unit (SICU) (17.79%). The Cardiac Vascular Intensive Care Unit (CVICU), Medical/Surgical Intensive Care Unit (M/SICU), Coronary Care Unit (CCU), and Neuro Surgical Intensive Care Unit (Neuro SICU) had 14.73%, 13.58%, 7.72%, and 7.06% of patients, respectively (Fig. 2).

### BMI and outcomes

**Fig. 3:**
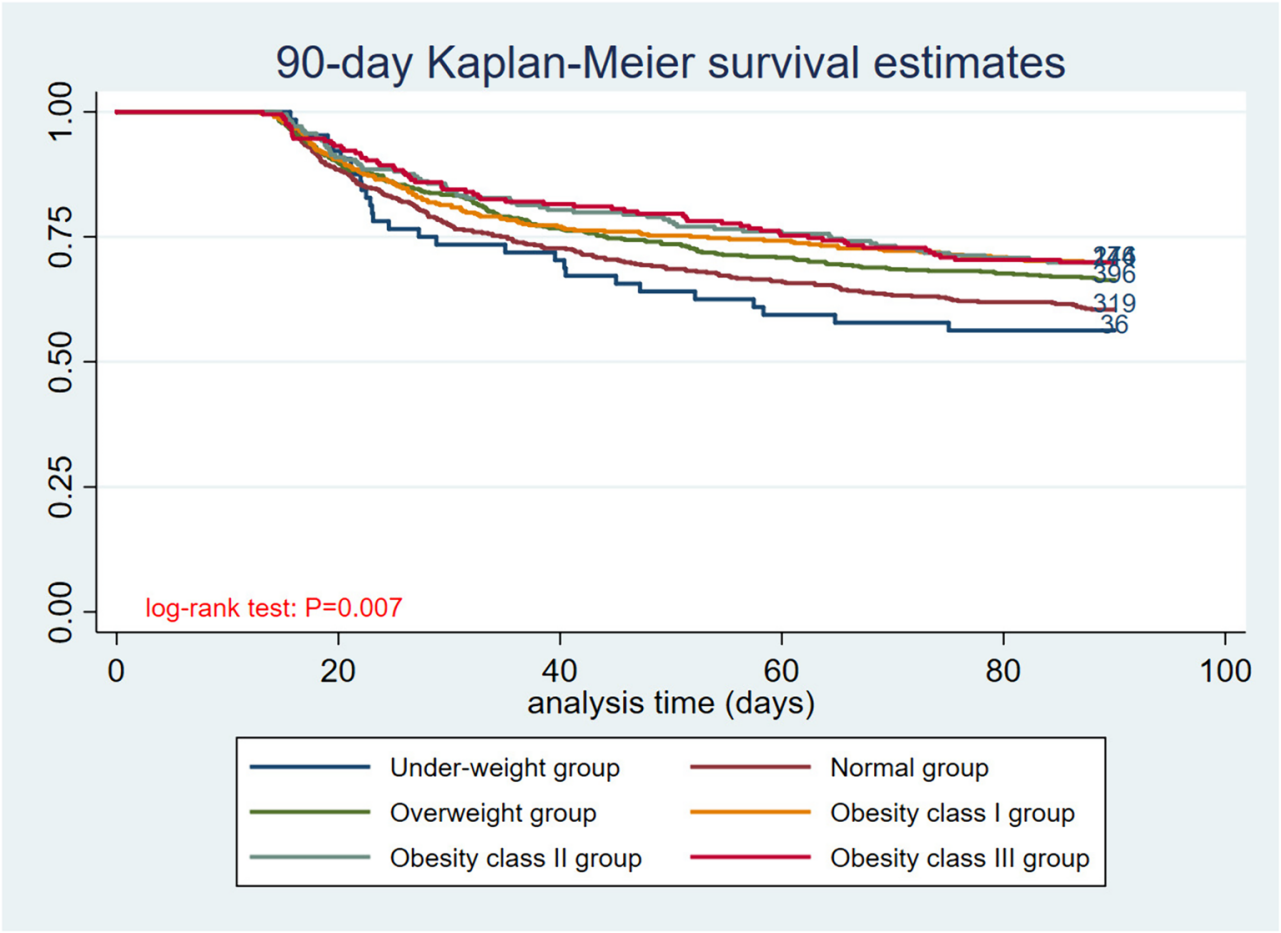
Kaplan–Meier curve for 90 day mortality by BMI category. The number at risk represents the number of patients in each BMI category

This study found that 90 day mortality varied significantly among different BMI groups. The underweight group showed the highest mortality of 43.75%, which declined gradually with increased BMI. The 90 day mortality in the obesity group was lowest, among which obesity class I group, obesity class II group, obesity class III group were 30.10%, 30.14%, 30.09%, respectively. After plotting the Kaplan–Meier curve of the 90 day mortality, the log-rank test showed that the difference was statistically significant (Fig. 3, *p* < 0.001).

Table 2 shows the patient prognoses of all study participants. The obesity group had the lowest 1 year mortality; this difference was statistically significant (*p* < 0.05). However, there were no statistically significant differences in length of ICU stay, length of hospital stay, ICU mortality, hospital mortality and 28 day mortality (*p* > 0.05).

**Table 2:** Outcomes of patients based on BMI categories

Variables	Under-weight group (n = 64, 3.21%)	Normal group (n = 528, 26.45%)	Overweight group (n = 597, 29.91%)	Obesity class I group (n = 392, 19.64%)	Obesity class II group (n = 209, 10.47%)	Obesity class III group (n = 206, 10.32%)	*P* value
Length of ICU stay (days)	20.72 (16.91–24.77)	19.85 (16.54–26.29)	20.83 (16.91–26.75)	20.52 (16.81–26.54)	20.54 (16.95–26.51)	21.16 (17.12–27.91)	0.387
Length of hospital stay (days)	25.00 (20.75–39.95)	26.56 (20.87–37.93)	28.75 (21.87–39.62)	27.75 (21.31–39.12)	28.25 (21.83–38.95)	28.45 (22.08–35.62)	0.297
ICU mortality, n (%)	14 (21.87)	108 (20.45)	120 (20.10)	72 (18.37)	35 (16.75)	34 (16.50)	0.685
Hospital mortality, n (%)	24 (37.5)	132 (25)	139 (23.28)	83 (21.17)	46 (22.01)	40 (19.42)	0.051
28 day mortality, n (%)	16 (25.00)	107 (20.25)	96 (16.08)	70 (17.86)	29 (13.88)	29 (14.08)	0.082
90 day mortality, n (%)	28 (43.75)	209 (39.58)	201 (33.66)	118 (30.10)	63 (30.14)	62 (30.09)	0.009
1 year mortality, n (%)	40 (62.5)	273 (51.70)	267 (44.72)	154 (39.29)	89 (42.58)	84 (40.78)	< 0.001

Identification of independent prognostic factors of 90 day mortality:

Using univariate Cox regression analysis, the results indicated that age, gender, Admission types, BMI, hemoglobin, RDW, creatinine, BUN, PT, APTT, INR, sodium, chloride, PaO2, anion gap, bicarbonate, lactate, heart rate, temperature, MBP, SPO2, congestive heart failure, COPD, hypertension, diabetes, Charlson comorbidity index, SAPSII, SOFA scores, sepsis, CRRT were significantly associated with 90 day mortality (Table 3, *p* < 0.05). Stepwise Cox regression analysis showed that age, gender, admission type, BMI, heart rate, MBP, temperature, chloride, RDW, PaO2, PaCO2, bicarbonate, charlson comorbidity index, CRRT were independent significantly associated with 90 day mortality in patients with CCI (*p* < 0.05).

**Table 3:** Univariate Cox regression analysis of 90 day mortality in CCI patients

Variables	HR	95%CI	*P* value
Age (years)	1.02	1.02–1.03	< 0.001
Male n (%)	0.80	0.68–0.93	0.004
White	0.95	0.81–1.11	0.529
Admission types			
Emergency	reference	reference	reference
Urgent	1.24	1.04–1.49	0.016
Surgery	1.16	0.95–1.42	0.125
Selected	0.52	0.34–0.79	0.003
BMI	0.98	0.97–0.99	0.001
Hemoglobin (g/dl)	0.91	0.88–0.93	< 0.001
WBC(× 10^9^/L)	1.00	0.99–1.01	0.438
PLT(× 10^9^/L)	0.99	0.99–1.00	0.055
RDW(%)	1.11	1.08–1.14	< 0.001
Creatinine (mg/dl)	1.08	1.04–1.13	< 0.001
BUN (mg/dL)	1.01	1.01–1.02	< 0.001
PT (S)	1.01	1.01–1.02	< 0.001
APTT (S)	1.00	1.00–1.01	0.003
INR	1.09	1.05–1.13	< 0.001
Potassium (mmol/L)	1.08	0.99–1.17	0.060
Sodium (mmol/L)	0.97	0.96–0.98	< 0.001
Chloride (mmol/L)	0.97	0.96–0.98	< 0.001
Calcium (mg/dL)	1.03	0.97–1.09	0.575
Glucose (mg/dL)	1.00	0.99–1.00	0.426
pH	0.39	0.21–0.74	0.004
PaO2 (mmHg)	0.99	0.98–0.99	0.003
PaCO2 (mmHg)	1.00	1.00–1.01	0.226
Anion gap (mmol/L)	1.02	1.01–1.04	0.002
Bicarbonate (mmol/L)	0.98	0.97–0.99	0.038
Lactate (mmol/L)	1.05	1.02–1.08	< 0.001
Heart rate (beats/minute)	1.00	1.00–1.01	0.029
Respiratory rate (beats/minute)	1.01	0.99–1.02	0.099
Temperature (°C)	0.88	0.82–0.94	< 0.001
MBP (mmHg)	0.98	0.98–0.99	< 0.001
SPO_2_ (%)	0.98	0.96–0.99	0.005
COPD	1.39	1.08–1.78	0.010
Congestive heart failure	1.66	1.29–2.15	< 0.001
Diabetes	1.27	1.08–1.48	< 0.001
Hypertension	0.88	0.75–1.03	0.125
Charlson comorbidity index	1.16	1.13–1.19	< 0.001
SAPS II	1.02	1.01–1.03	< 0.001
SOFA	1.06	1.04–1.08	< 0.001
Sepsis	2.02	1.73–2.35	< 0.001
CRRT	1.75	1.49–2.05	< 0.001
Ventilation	0.96	0.65–1.41	0.857
Vasoactive agent	1.66	1.33–2.07	< 0.001

This study used multivariable Cox regression analysis to determine the relationship between BMI and the 90 day mortality of patients with CCI. In Model I, no parameters were adjusted, and in Model II, only gender and age were adjusted. Model III was a completely adjusted model that controlled for all potential confounders and showed a statistically significant association between higher BMI and decreased mortality risk (HR (95% CI): 0.97 (0.96–0.99), *p* < 0.001). The mortality risk of the overweight group and the obesity group were lower than those of the normal group, with statistically significant differences (*p* < 0.05) (Table 4).

**Table 4:** Multivariable Cox regression analysis for 90-day mortality

	Model I		Model II		Model III	
Varables	HR(95%CI)	*p* value	HR(95%CI)	*p* value	HR(95%CI)	*p* value
BMI	0.98 (0.97–0.99)	0.001	0.98 (0.97–0.99)	< 0.001	0.97 (0.96–0.99)	< 0.001
Under-weight	1.13 (0.76–1.68)	0.52	1.15 (0.77–1.71)	0.479	1.07 (0.72–1.61)	0.710
Normal	reference	reference	reference	reference	reference	reference
Overweight	0.81 (0.67–0.98)	0.039	0.82 (0.67–1.00)	0.052	0.73 (0.60–0.89)	0.002
Obesity class I group	0.72 (0.57–0.91)	0.005	0.72 (0.57–0.91)	0.005	0.67(0.53–0.84)	0.001
Obesity class II group	0.71 (0.53–0.93)	0.016	0.66 (0.50–0.88)	0.005	0.55(0.41–0.74)	< 0.001
Obesity class III group	0.70 (0.52–0.93)	0.015	0.66 (0.50–0.88)	0.005	0.61(0.45–0.81)	< 0.001

Model I adjusted for none,

Model II adjusted for age, gender, race and admission types,

Model III adjusted for 41 potential variables included demographic characteristics, laboratory parameters, blood gas, vital signs, comorbidities, severity scoring systems, organ support and diagnosis by stepwise Cox regression. The result shows that age, gender, admission type, BMI, heart rate, MBP, temperature, chloride, RDW, PaO2, PaCO2, bicarbonate, charlson comorbidity index, CRRT were influence factor of 90-day mortality in patients with CCI

**Fig. 4:**
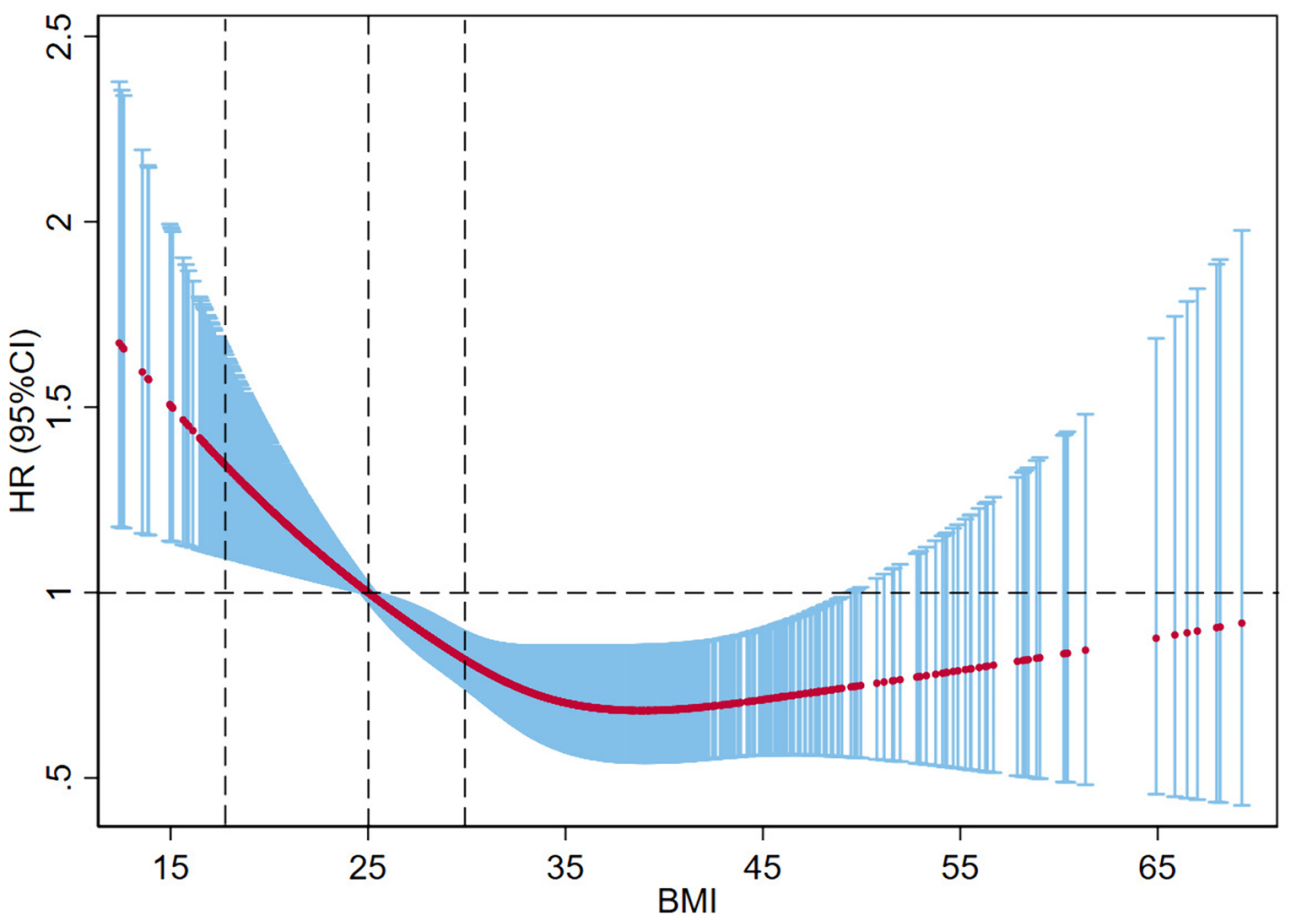
The association between BMI and 90 day mortality in the CCI patients after adjusting for confounding factors. Dashed vertical lines represent WHO BMI category thresholds of 18.5 kg/m^2 (underweight to healthy), 25 kg/m^2 (healthy weight to overweight), and 30 kg/m^2 (overweight to obese)

Using a multivariable restricted cubic spline, this study investigated the relationship between BMI as a continuous variable and 90 day mortality in patients with CCI. The results showed a non-linear (inverse J-shaped) relationship between BMI and 90 day mortality and a negative correlation when BMI was less than 38 kg/m^2. The higher the BMI, the lower the risk of death was observed in CCI patients (Fig. 4).

### Subgroup analyses and interaction analysis

Subgroup analysis was conducted to further investigate the relationship between BMI and 90 day mortality of patients with CCI in different stratifications. The results revealed an interaction between BMI and age (Table 5, *p* = 0.010).

**Table 5:** A subgroup analysis for the effect of obesity on the risk of 90-day mortality after adjusting for all confounding factors

Variables	N	HR (95% CI)	*P* value	*P* for interaction
Age				0.010
≤ 65	978	0.98 (0.97–1.01)	0.147	
> 65	1018	0.97 (0.95–0.99)	0.000	
Gender				0.941
Female	812	0.97 (0.96–0.99)	0.002	
Male	1184	0.97 (0.96–0.99)	0.002	
Admission type				0.809
Emergency	1048	0.97 (0.96–0.99)	0.004	
Urgent	464	0.97 (0.95–0.99)	0.025	
Surgery	361	0.97 (0.94–0.99)	0.044	
Selected	123	0.95 (0.89–1.02)	0.183	
RDW				0.953
≤ 14.7	989	0.97 (0.95–0.99)	0.011	
> 14.7	1007	0.97 (0.96–0.98)	0.000	
Chloride				0.615
≤ 103	1060	0.97 (0.96–0.99)	0.001	
> 103	936	0.98 (0.96–0.99)	0.017	
PO2				0.946
≤ 104.5	988	0.98 (0.97–0.99)	0.006	
> 104.5	1008	0.97 (0.95–0.98)	0.001	
PCO2				0.297
≤ 41	1062	0.98 (0.96–0.99)	0.048	
> 41	934	0.97 (0.95–0.98)	0.000	
Bicarbonate				0.772
≤ 22	1036	0.98 (0.96–0.99)	0.029	
> 22	960	0.96 (0.95–0.98)	0.000	
Temperature				0.241
≤ 36.73	961	0.97 (0.96–0.99)	0.002	
> 36.73	1035	0.98 (0.96–0.99)	0.003	
MBP				0.146
≤ 82	1028	0.97(0.96–0.98)	0.000	
> 82	968	0.97(0.96–0.99)	0.007	
Heart rate				0.928
≤ 91	1026	0.97(0.95–0.99)	0.002	
> 91	970	0.97(0.96–0.99)	0.005	
Charlson comorbidity index				0.106
≤ 6	1174	0.97 (0.96–0.99)	0.005	
> 6	822	0.98 (0.96–0.99)	0.007	
Vasoactive agent				0.500
No	379	0.99 (0.96–1.02)	0.516	
Yes	1617	0.97 (0.96–0.99)	0.000	
CRRT				0.895
No	1496	0.97 (0.96–0.98)	0.000	
Yes	500	0.97 (0.96–0.99)	0.021	

**Fig. 5:**
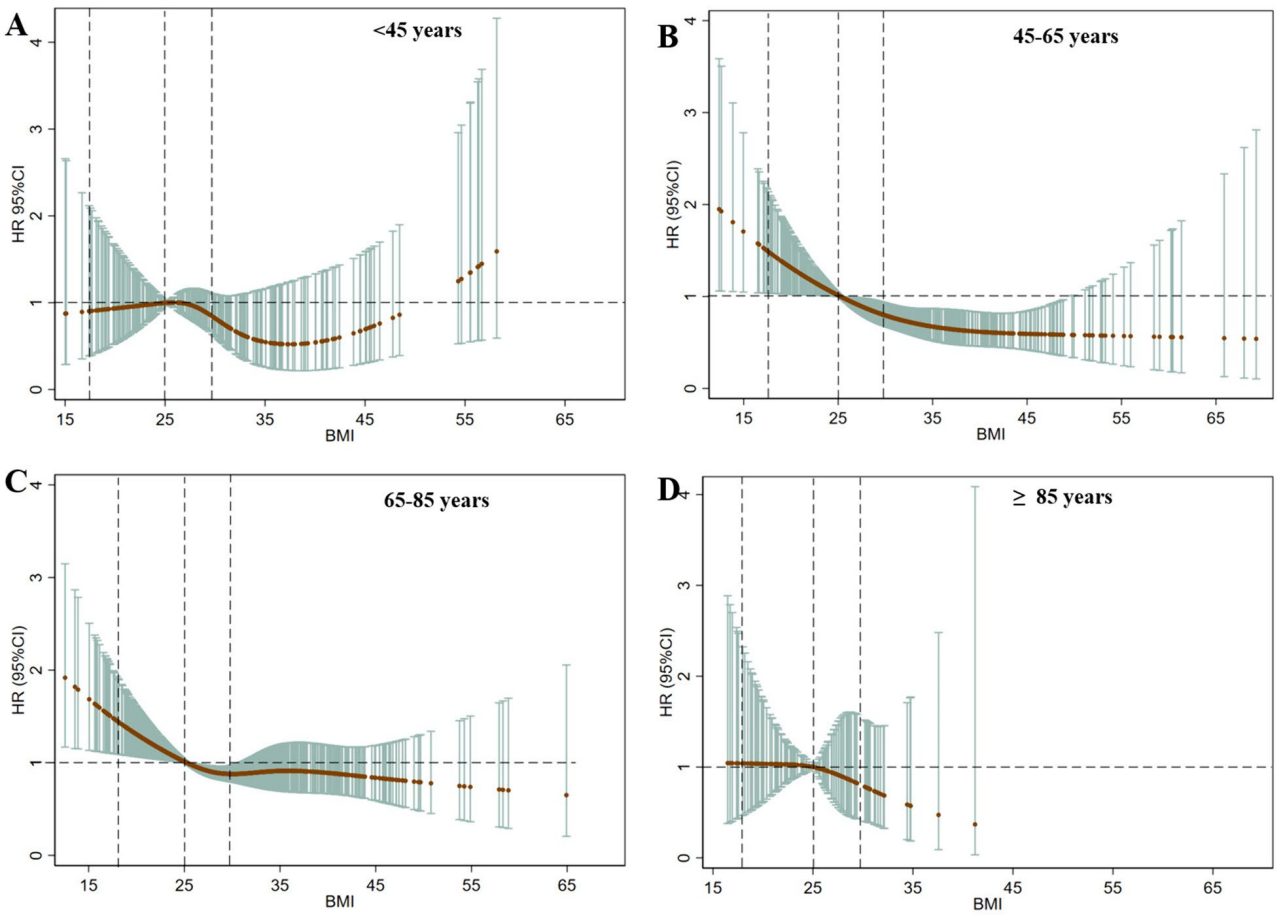
The association between BMI and 90 day mortality in CCI patients according to age categories Dashed vertical lines represent WHO BMI category thresholds of 18.5 kg/m^2 (underweight to healthy), 25 kg/m^2 (healthy weight to overweight), and 30 kg/m^2 (overweight to obese)

The present study found an interaction between BMI and age. We plotted the multivariable restricted cubic splines to further understand the relationship between BMI and 90 day mortality among patients with CCI in different age categories (Fig. 5). The results showed that elderly (65–85 years old) and very elderly (≥ 85 years old) patients from the obesity group had a more distinct survival advantage, but the proportion of obesity in very elderly CCI patients was small. However, patients younger than 45 were the only subgroup which showed survival advantage in BMI < 25 kg/m^2. It also had an obvious BMI turning point at 38 kg/m^2, beyond which the risk of mortality increased with increasing BMI (See Fig. 5A).

The association between BMI and 90 day mortality in CCI patients who survived after discharge:

To further study the long-term prognosis of CCI patients with different BMIs, we excluded the patients who died in the hospital (n = 464). The study flowchart is shown in supplement Fig. 1. After excluding patients who died in the hospital, 1532 patients were included and divided into six groups based on the WHO BMI classification. Notably, the proportion of patients in each group did not change even after excluding patients who died in the hospital. The general characteristics, laboratory parameters, blood gas, vital signs, comorbidities, disease severity scores, and prognosis of the six groups are shown in supplement Table 1. After plotting the Kaplan–Meier curve of the 90 day mortality, a log-rank test showed that the difference was statistically significant (supplement Fig. 2, *p* < 0.001). The prognoses of all participants are shown in supplement Table 2. The obesity group had the lowest 90 day mortality, 1 year mortality, and these differences were statistically significant (*p* < 0.05). However, there were no statistically significant differences in length of ICU stay and length of hospital stay (*p* > 0.05). The results of univariate Cox regression analysis are shown in supplement Table 3.

We also performed different statistical models to verify the stability of the results. Model III was a completely adjusted model that accounted for all potential confounders, and it demonstrated a statistically significant association between higher BMI and decreased mortality risk (HR (95% CI): 0.97 (0.95–0.99), *P* = 0.008). The mortality risk of the overweight group and the obesity group were lower than those of the normal group, with statistically significant differences (*P* < 0.05) (supplement Table 4). The multivariable restricted cubic spline showed a non-linear (inverse J-shaped) relationship between BMI and 90 day mortality (supplement Fig. 3).

## Discussion

The present study found that the 90 day mortality of patients with CCI was as high as 34.12%, and the 1 year mortality was 45.44%. Our results found that the incidence of hypertension and diabetes in obese patients was significantly higher than in non-obese patients; however, obesity was associated with a lower 90 day mortality. After adjusting for potential confounders, the mortality risk of overweight (BMI 25–30 kg/m^2) and obese (BMI ≥ 30 kg/m^2) CCI patients was significantly lower compared to patients with an average BMI (18.5–25 kg/m^2), confirming the existence of the obesity paradox in patients with CCI. Interestingly, the results were consistent after the exclusion of patients who died in hospital. Our results were consistent with previous studies. Li et al. have reported that the obesity paradox applied to both all-cause and cause-specific mortality among critically ill men and women, as demonstrated in a large cohort study [33].

The prognosis of patients with CCI is poor, with less than 10% regaining independent living ability [34]. Iwashyna et al.’s study showed that although patients with CCI only accounted for 5% of ICU admissions, they attributed to more than 30% of ICU hospitalization days and 14% of total hospitalization days [35], posing a significant burden on families and society.

Given the rise in the prevalence of obesity due to socioeconomic developments, its presence in ICU has become increasingly common. It has been found that nearly one-third of ICU patients reach the obesity standard, and up to 7% are morbidly obese [36, 37]. Given the significant number of patients, it is crucial to understand the role of obesity in critically ill patients. Our findings showed that the proportion of CCI patients who were overweight or obese was as high as 29.91% and 40.43%, respectively. These data not only validate previous studies, but also raise pressing concerns regarding the management of obese patients in ICU, particularly with CCI. The high prevalence of obesity among CCI patients necessitates researchers to investigate tailored care strategies and emphasizes the need for healthcare systems to adjust treatments accordingly to adapt to this growing challenge.

CCI is a highly heterogeneous disease resulted from multiple acute critical illness [38]. Sepsis was the most likely diseases to develop CCI, our results showed that patients with sepsis accounted for 39.63% of CCI. On the other hand, CCI is also prevalent among patients in surgical ICUs (SICU and TSICU), indicating that patients who undergo surgery are at a higher risk for developing CCI. Therefore, it is crucial for healthcare providers to closely monitor and prevent the development of CCI in these patients to improve their overall outcomes.

The question of why a high BMI leads to lower mortality in patients with CCI remains intriguing. At present, we hypothesize that the potential mechanisms in CCI could involve: (1) Higher energy reserves: During critical periods, excess fat cells in obese patients can enhance glucose absorption and metabolism, potentially reducing the risk of hypoglycemia. This lower risk of hypoglycemia may contribute to decreased mortality and could partly elucidate the obesity paradox observed in these patients [39]. (2) Adipose tissue might effectively diminish the levels of potentially detrimental factors like TNF-α, thereby mitigating their impact in patients with CCI [40]; (3) It has been suggested by some researchers that due to the general medical perception associating obesity with adverse outcomes, obese patients with CCI might receive more intensive treatment, ultimately resulting in improved prognoses [22]. Additionally, patients with CCI often necessitate extended ICU stays, are in a state of heightened catabolism, and face a significant risk of muscle wasting and weakness, contributing to poorer prognoses. It has been established that premorbid obesity aids in the efficient use of stored lipids and mitigates muscle wasting and weakness in sepsis and critically ill patients [41]. Consequently, obese patients with CCI may derive benefits from this attenuation of muscle wasting and weakness. Furthermore, obese patients typically experience chronic, low-grade inflammation. Simultaneously, patients with CCI undergo a mild yet persistent state of inflammation and immunosuppression. Obese patients have been found to tolerate ongoing inflammatory stimuli better than their normal-weight counterparts [12, 42–44]. This might account for the lesser vulnerability of obese patients to adverse outcomes compared to non-obese patients with CCI. These potential mechanisms collectively suggest that obese patients with CCI have a survival advantage.

Recent findings have linked obesity with a reduced mortality risk in critically ill patients [25, 39, 45]. In cases involving extracorporeal membrane oxygenation (ECMO) for acute respiratory distress syndrome (ARDS), obese patients have been observed to exhibit lower ICU mortality compared to non-obese patients, as confirmed through multivariable analysis and further supported by propensity score matching [46]. Studies have shown that ventilator-induced lung injury may be lessened in obese patients due to increased chest wall elastance, which provides a protective benefit by absorbing some transpulmonary pressure [47]. In our study, the utilization of mechanical ventilation was notably high, reaching 95.99%. This decrease in ventilator-induced lung injury could be a contributing factor to the favorable outcomes observed in obese patients with CCI. Additionally, obesity’s correlation with functional outcomes was noted in Asian patients with sepsis; the obese group exhibited lower frailty at discharge compared to the non-obese group [48]. These results further reinforce our conclusion that obesity may enhance patient outcomes in CCI by reducing the incidence of frailty.

However, the relationship between BMI and mortality in critical illness remains a subject of debate. A retrospective cohort study indicated an increased risk of 28-day mortality in obese patients with acute kidney injury undergoing continuous renal replacement therapy [49]. Our study, contrasting these findings, suggests that a high BMI is associated with lower mortality in patients with CCI. Nevertheless, potential biases and confounding factors in our study could influence these results [50]. After excluding in-hospital deaths, our findings also revealed a lower 90 day mortality among obese patients, effectively negating the presence of survivor bias in this group. This is a pivotal aspect in validating the accuracy of our study and supporting the existence of the obesity paradox in patients with CCI.

The subgroup analysis demonstrated an interaction between BMI and age, with lower mortality risk in people aged ≥ 65 years [HR (95% CI): 0.98 (0.97–1.01) vs. 0.97 (0.95–0.99)], which is consistent with the study of Zhou et al., who argued that being overweight or obese may benefit critically ill elderly patients [51]. However, a large cohort study that included 3.6 million adults found the correlation between higher BMI and lower mortality weakened with age [52], but the study population was non-critical. Another investigation focusing on critically ill COVID-19 patients revealed that a higher BMI was correlated with reduced mortality in individuals under 45 years of age. However, this outcome was ascribed to an as-yet unexplained biological mechanism, further complicated by the presence of confounding biases [45]. Our findings revealed that the association between BMI and 90 day mortality was more pronounced among elderly (65–85 years) and very elderly (≥ 85 years) patients with CCI. We speculated that elderly and very elderly patients were more likely to suffer weight loss, muscle loss, and functional decline. In contrast, obese patients may benefit from having greater nutritional reserves, which could provide a protective effect. In addition, high BMI was a protective factor of sarcopenic, which decreased the risk of all-cause mortality. However, sarcopenic obesity was associated with increased mortality in elderly patients [53].

To the best of our knowledge, this is the first study to report the obesity paradox phenomenon in patients with CCI. Notably, we found this phenomenon was more pronounced among elderly and very elderly patients. More clinical studies are necessary to be performed to reveal the inner connection of obesity paradox phenomenon in patients with CCI and to explain the difference between the subgroups with different age.

## Limitations

(1). Due to its retrospective observational design, this study may have an insufficient adjustment for confounding variables and selection bias, which could lead to inaccurate associations between obesity and mortality. However, we conducted three statistical models to verify the consistency of the results. (2). Body fat mass and fat distribution were identified as important roles in explaining the observed obesity paradox. However, we selected BMI as the obesity index despite its limitations in accurately reflecting body fat mass and fat distribution compared to waist circumference and waist-hip ratio. This limitation could overestimate the degree of obesity in patients with higher muscle mass and underestimate the extent of obesity in elderly patients with CCI. (3). Sarcopenic obesity is an important factor that was not included in the data, which could potentially overestimate the outcome. Therefore, further large prospective studies utilizing waist circumference and waist-hip ratio categories are needed to validate the relationship between obesity and mortality in CCI patients.

## Conclusion

Patients with CCI have a high reoccurrence rate and poor prognosis. We found that high BMI was associated with lower mortality, and this relationship was influenced by age. These findings were more pronounced among elderly (65–85 years) and very elderly (≥ 85 years) patients, indicating the existence of the obesity paradox among patients with CCI. Future research should investigate whether the obesity paradox is caused by adipose-related biological mechanisms and utilize more accurate weight indicators than BMI. Furthermore, healthcare providers must reconsider their strategies for preventing and treating obesity among patients with CCI based on the present findings.

### Supplementary Information

Supplementary tables and figures.

## Data Availability

All authors declare that raw data and other materials are available upon requested.

## Declarations

**Ethical approval and consent to participate.**

Data for this study was sourced exclusively from the MIMIC-IV database. As this was a retrospective analysis using de-identified patient data, no ethical concerns or issues related to clinical trial participants were implicated. No additional institutional review board approval was required for this analysis.
