# Supplementary material for: The obesity paradox and 90 day mortality in chronic critically ill patients: a cohort study using a large clinical database
Source: Eur J Med Res. 2024 Jul 29;29:392. doi: 10.1186/s40001-024-01962-w (PMC11285416; doi:10.1186/s40001-024-01962-w)

**Supplemental Figure 1: Study Flowchart for CCI Patients Who Survived Post-Discharge.**

**
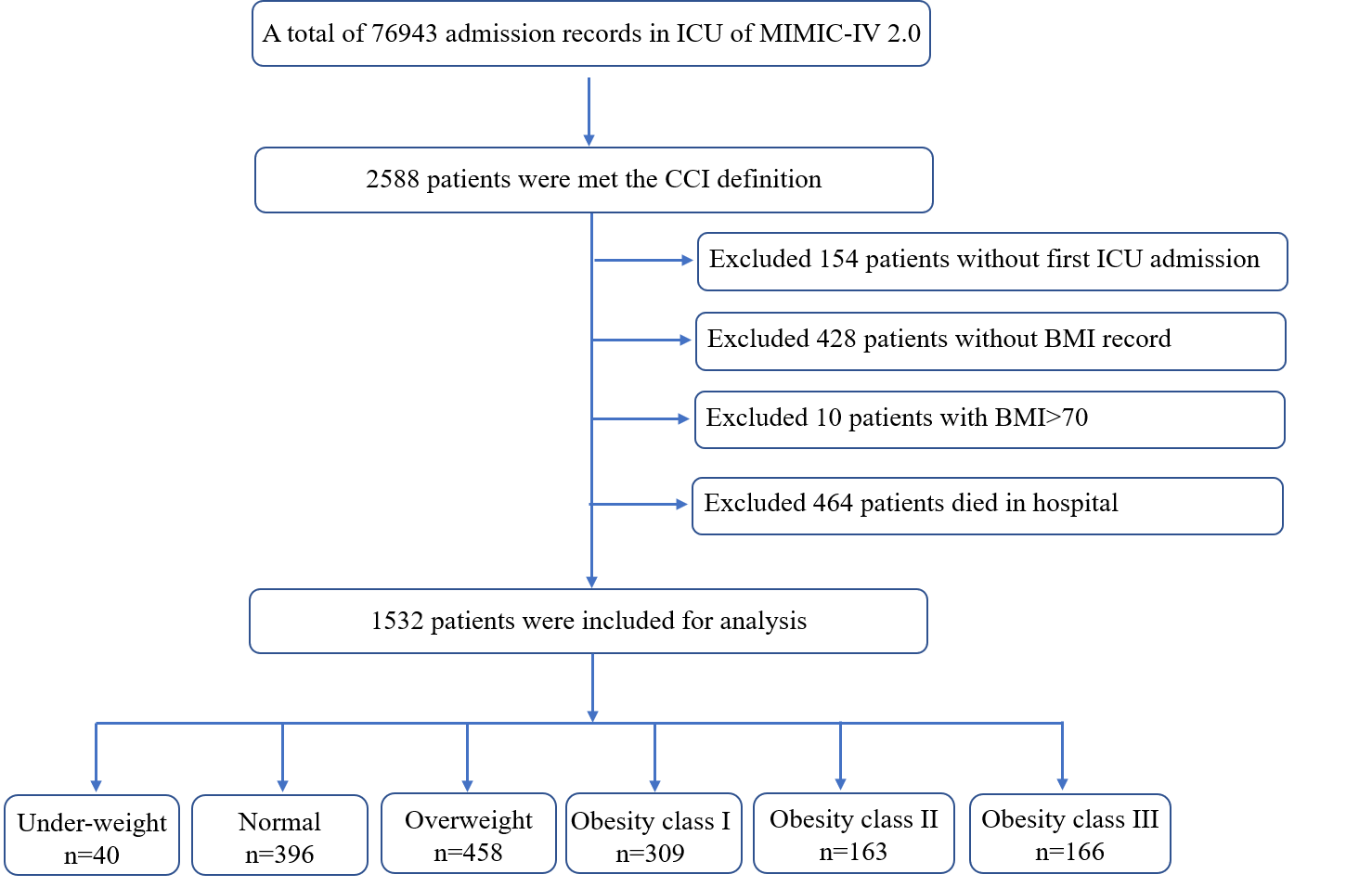
**

**Supplemental Table 1: Characteristics of Patients Who Survived Post-Discharge, Categorized by BMI.**

| **Variables** | **under-weight group**  **(n=40, 2.61%)** | **Normal group**  **(n=396, 25.85%)** | **overweight group**  **(n=458, 29.90% )** | **Obesity class I group**  **(n=309, 20.16%)** | **Obesity class II group**  **(n=163, 10.64%)** | **Obesity class III group**  **(n=166, 10.84)** | **p-value** |
| --- | --- | --- | --- | --- | --- | --- | --- |
| **Demographic characteristics** |  |  |  |  |  |  |  |
| Age (years) | 64.95±18.29 | 63.08±17.48 | 62.80±15.82 | 62.04±14.88 | 62.24±14.81 | 59.61±13.49 | 0.001 |
| Male n (%) | 22 (55.00) | 226 (57.07) | 309 (67.47) | 209 (67.67) | 85 (52.15) | 78 (46.99) | < 0.001 |
| White n (%) | 19 (1.98) | 236 (24.61) | 300 (31.28) | 206 (21.48) | 92 (9.59) | 106 (11.06) | 0.035 |
| **Admission Type** |  |  |  |  |  |  | 0.007 |
| Emergency | 23 (2.80) | 217 (26.43) | 256 (31.18) | 160 (19.49) | 75 (9.14) | 90 (10.96) |  |
| Urgent | 7 (2.06) | 68 (20.00) | 89 (26.18) | 73 (21.47) | 52 (15.29) | 51 (15.00) |  |
| Surgery | 7 (2.62) | 82 (30.71) | 81 (30.34) | 55 (20.60) | 27 (10.11) | 15 (5.62) |  |
| Selected | 3 (2.88) | 29 (27.88) | 32 (30.77) | 21 (20.19) | 9 (8.65) | 10 (9.63) |  |
| **Laboratory parameters** |  |  |  |  |  |  |  |
| Hemoglobin (g/dl） | 11.20±2.68 | 10.94±2.51 | 11.26±2.68 | 11.27±2.75 | 10.94±2.72 | 11.63±2.64 | 0.640 |
| WBC(×10^9^/L) | 11.25 (8.60-15.60) | 11.1 (8.0-15.6) | 12.2 (8.1-17.0) | 11.95 (8.05-16.80) | 12.10 (8.80-17.20) | 12.85 (8.30-17.20) | 0.325 |
| PLT(×10^9^/L) | 218 (150-308) | 197 (132-270) | 202 (143-274.00) | 195.50 (129.50-247.00) | 208.00(152.00-269.00) | 214.00(152.00-288.00) | 0.044 |
| RDW (%) | 15.32±2.33 | 14.87±2.33 | 15.12±2.57 | 15.01±2.33 | 15.49±2.46 | 15.60±2.33 | < 0.001 |
| Creatinine (mg/dl) | 0.85 (0.65-1.50) | 0.90 (0.70-1.30) | 1.10(0.80-1.60) | 1.20 (0.80-1.90) | 1.30 (0.90-2.20) | 1.20 (0.80-1.90) | < 0.001 |
| BUN (mg/dL) | 21.00 (13.00-30.50) | 17.00 (12.00-29.00) | 20.00(14.00-32.50) | 21.50 (15.00-38.50) | 24.00 (15.00-41.00) | 23.00 (16.00-38.00) | < 0.001 |
| PT(S) | 13.00 (11.95-15.00) | 13.60(12.20-16.60) | 13.70(12.30-17.20) | 14.20 (12.50-17.60) | 13.70 (12.40-16.90) | 13.95 (12.60-17.00) | 0.046 |
| APTT(S) | 31.45 (28.45-35.80) | 30.40 (27.10-38.20) | 30.75 (26.60-38.60) | 31.50 (26.80-39.90) | 30.60 (26.20-37.30) | 30.10 (26.40-37.90) | 0.748 |
| INR | 1.20 (1.10-1.30) | 1.20(1.10-1.50) | 1.20 (1.10-1.60) | 1.30 (1.10-1.60) | 1.20 (1.10-1.60) | 1.30 (1.20-1.60) | 0.082 |
| Potassium (mmol/L) | 4.57±1.05 | 4.13±0.84 | 4.27±0.81 | 4.31±0.86 | 4.40±0.90 | 4.55±1.01 | 0.005 |
| Sodium (mmol/L) | 140.92±7.60 | 138.59±5.64 | 138.25±5.78 | 138.57±5.49 | 138.61±5.58 | 138.41±5.33 | 0.064 |
| Chloride (mmol/L) | 105.40±8.05 | 103.75±7.44 | 103.10±7.43 | 103.11±6.79 | 102.92±7.06 | 101.94±6.81 | 0.316 |
| Calcium (mg/dL) | 8.40±0.90 | 8.29±1.03 | 8.31±1.01 | 8.32±1.02 | 8.25±1.07 | 8.30±1.01 | 0.824 |
| Glucose(mg/dL) | 116.00 (104.00-149.00) | 133.00(110.00-172.00) | 133.50 (110.00-174.00) | 146.50(112.50-190.50) | 140.00 (114.00-174.00) | 149.50 (114.00-190.00) | 0.001 |
| **Blood gas** |  |  |  |  |  |  |  |
| pH | 7.33 (7.27-7.43) | 7.38 (7.31-7.43) | 7.38 (7.29-7.44) | 7.37 (7.28-7.42) | 7.35 (7.27-7.40) | 7.35 (7.28-7.41) | < 0.001 |
| PaO2(mmHg) | 122.00 (66.00-194.00) | 138.00 (70.00-252.00) | 112.00 (70.00-194.00) | 105.00 (73.00-191.00) | 90.00 (65.00-167.00) | 88.50 (62.00-123.00) | < 0.001 |
| PaCO2(mmHg) | 44.00 (37.00-63.00) | 40.00 (34.00-45.00) | 39.00 (34.00-47.00) | 41.00 (35.00-48.00) | 44.00 (37.00-52.00) | 45.50 (38.00-55.00) | < 0.001 |
| Anion gap(mmol/L) | 14.00 (12.00-17.50) | 15.00 (12.00-18.00) | 15.50 (13.00-19.00) | 16.00 (13.00-19.00) | 15.00 (13.00-18.00) | 15.00 (13.00-19.00) | 0.042 |
| Bicarbonate (mmol/L) | 22.50 (20.00-27.00) | 22.00 (19.00-25.00) | 22.00 (19.00-25.00) | 22.00 (19.00-26.00) | 22.00 (19.00-26.00) | 24.00 (20.00-27.00) | 0.086 |
| Lactate (mmol/L) | 1.30 (1.00-2.50) | 1.60 (1.10-2.60) | 1.70 (1.20-2.90) | 1.80 (1.20-3.10) | 1.90 (1.30-3.20) | 1.80 (1.20-2.75) | 0.009 |
| **vital signs** |  |  |  |  |  |  |  |
| Heart rate (beats/minute) | 91.87±20.72 | 91.71±21.16 | 91.07±21.51 | 93.50±21.93 | 91.65±23.55 | 93.24±20.39 | 0.520 |
| Respiratory rate (beats/minute) | 19.60±6.25 | 20.43±6.69 | 20.69±6.42 | 20.91±6.24 | 20.99±7.08 | 21.95±6.02 | 0.282 |
| Temperature (°C) | 36.54±0.94 | 36.72±0.93 | 36.73±1.11 | 36.85±1.11 | 36.81±1.28 | 36.89±0.81 | < 0.001 |
| MBP (mmHg) | 85.00 (71.00-97.50) | 83.00 (72.00-97.00) | 83.00 (71.00-96.00) | 82.00 (71.00-97.00) | 81.00 (68.00-95.00) | 81.00 (68.00-95.00) | 0.386 |
| SPO2 (%) | 99.00 (97.00-100.00) | 99.00 (96.00-100.00) | 98.00 (95.00-100.00) | 98.00 (94.00-100.00) | 98.00 (95.00-100.00) | 96.00 (93.00-99.00) | < 0.001 |
| **Comorbidities, n (%)** |  |  |  |  |  |  |  |
| COPD | 5 (12.50) | 25 (6.31) | 17 (3.71) | 26 (8.41) | 17 (10.43) | 22 (13.25) | < 0.001 |
| Congestive heart failure | 2 (5.00) | 20 (5.05) | 24 (5.24) | 22 (7.12) | 10 (6.13) | 13 (7.83) | 0.720 |
| Diabetes | 5 (12.50) | 72 (18.18) | 124 (27.07) | 121 (39.16) | 71 (43.56) | 81 (48.8.) | < 0.001 |
| Hypertension | 12 (30.00) | 141 (35.60) | 170 (37.12) | 140 (45.31) | 66 (40.49) | 63 (37.95) | 0.097 |
| Charlson comorbidity index | 5.50 (3.50-8.50) | 5.00 (4.00-7.00) | 6.00 (4.00-8.00) | 6.00 (4.00-8.00) | 6.00 (4.00-8.00) | 5.00 (4.00-7.00) | 0.495 |
| **Severity Scoring systems** |  |  |  |  |  |  |  |
| SAPS II | 41.50 (34.00-54.50) | 40.00 (31.00-51.00) | 40.00 (31.00-52.00) | 42.00 (33.00-51.00) | 42.00 (34.00-54.00) | 41.00 (32.00-48.00) | 0.178 |
| SOFA | 8.00 (4.00-11.00) | 8.00 (5.00-11.00) | 8.00 (5.00-12.00) | 9.00 (6.00-13.00) | 10.00 (7.00-13.00) | 9.00 (7.00-12.00) | < 0.001 |
| **Organ support, n (%)** |  |  |  |  |  |  |  |
| CRRT | 2 (5.00) | 56 (14.14) | 103 (22.49) | 66(21.36) | 52 (31.90) | 42 (25.30) | < 0.001 |
| Invasive ventilation | 38 (95.00) | 381 (96.21) | 436 (95.20) | 297 (96.12) | 160 (98.16) | 158 (95.18) | 0.676 |
| Vasoactive agent | 29 (72.50) | 310 (78.28) | 361 (78.82) | 246 (79.61) | 131 (80.37) | 129 (77.71) | 0.915 |
| **Sepsis n (%)** | 15 (37.5) | 118 (29.79) | 148 (32.31) | 105 (33.98) | 52 (31.90) | 77 (46.39) | 0.008 |

Note:S: seconds; MBP: Mean blood pressure;pH: power of hydrogen; PaO2: Arterial partial pressure of oxygen PaCO2:Arterial partial pressure of carbon dioxide;SPO2: Peripheral capillary oxygen saturation; COPD: Chronic obstructive pulmonary disease. Vasoactive agent included noradrenaline, vasopressin, phenylephrine, epinephrine, dopamine.

**Supplemental Figure 2: Kaplan-Meier Curve Representing 90-day Mortality among CCI Patients Who Survived Post-Discharge, Categorized by BMI. The 'number at risk' denotes the count of patients in each BMI category.**


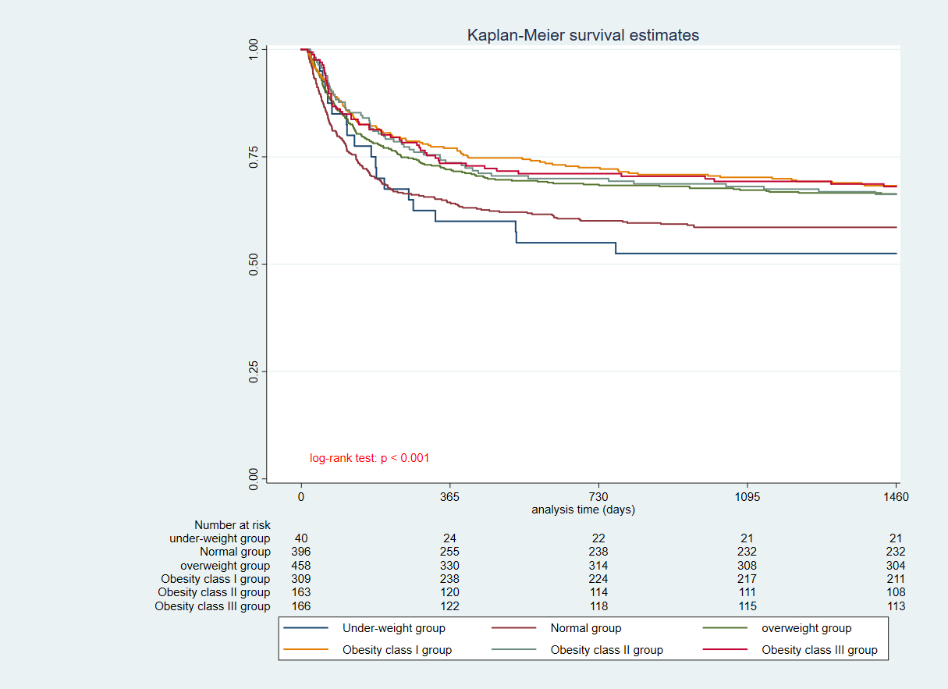


**Supplemental Table 2: Outcomes for CCI Patients Who Survived Post-Discharge, Stratified by BMI Categories.**

| **Variables** | **under-weight group**  **(n=40, 2.61%)** | **Normal group**  **(n=396, 25.85%)** | **overweight group**  **(n=458, 29.90% )** | **Obesity class I group**  **(n=309, 20.16%)** | **Obesity class II group**  **(n=163, 10.64%)** | **Obesity class III group**  **(n=166, 10.84)** | **p-value** |
| --- | --- | --- | --- | --- | --- | --- | --- |
| Length of ICU stay (days) | 20.56 (16.72-26.79) | 20.25 (16.79-26.29) | 21.25 (17.29-26.95) | 21.33 (16.95-27.20) | 20.58 (16.95-27.20) | 21.16 (17.54-27.91) | 0.415 |
| Length of hospital stay (days) | 27.20 (20.75-39.54) | 27.41 (21.95-39.89) | 29.83 (22.91-40.91) | 29.75 (23.08-42.00) | 28.95 (22.41-39.08) | 29.72 (22.83-36.04) | 0.252 |
| 90-day mortality, n (%) | 6 (15.00) | 80 (20.36) | 65 (14.19) | 35 (11.33) | 19 (11.66) | 23 (13.85) | 0.019 |
| 1-year mortality, n (%) | 16 (40.00) | 141(35.61) | 128 (27.95) | 71 (22.98) | 43 (26.38) | 44 (26.50) | 0.004 |

**Supplemental Table 3: Univariate Cox Regression Analysis of 90-day Mortality Among CCI Patients Who Survived Post-Discharge.**

| **Variables** | **HR** | **95%CI** | **P value** |
| --- | --- | --- | --- |
| Age (years) | 1.03 | 1.02-1.04 | < 0.001 |
| Male n (%) | 0.82 | 0.63-1.06 | 0.138 |
| White | 0.86 | 0.66-1.12 | 0.284 |
| Admission types |  |  |  |
| emergency | reference | reference | reference |
| urgent | 1.26 | 0.93-1.72 | 0.131 |
| surgery | 1.11 | 0.78-1.58 | 0.554 |
| selected | 0.38 | 0.16-0.86 | 0.021 |
| BMI | 0.97 | 0.95-0.99 | 0.010 |
| Hemoglobin (g/dl） | 0.89 | 0.85-0.94 | < 0.001 |
| WBC(×10^9^/L) | 1.00 | 0.99-1.01 | 0.837 |
| PLT(×10^9^/L) | 0.99 | 0.97-1.00 | 0.089 |
| RDW(%) | 1.11 | 1.06-1.16 | < 0.001 |
| Creatinine (mg/dl) | 1.08 | 1.01-1.16 | 0.013 |
| BUN (mg/dL) | 1.01 | 1.01-1.02 | < 0.001 |
| PT (S) | 1.01 | 0.99-1.02 | 0.112 |
| APTT (S) | 1.00 | 0.99-1.01 | 0.186 |
| INR | 1.06 | 0.97-1.16 | 0.162 |
| Potassium (mmol/L) | 0.99 | 0.86-1.15 | 0.972 |
| Sodium (mmol/L) | 0.96 | 0.94-0.98 | 0.002 |
| Chloride (mmol/L) | 0.97 | 0.95-0.98 | 0.002 |
| Calcium (mg/dL) | 1.01 | 0.89-1.14 | 0.881 |
| Glucose (mg/dL) | 1.00 | 0.99-1.00 | 0.935 |
| pH | 0.63 | 0.20-2.01 | 0.443 |
| PaO2 (mmHg) | 0.99 | 0.99-1.00 | 0.116 |
| PaCO2 (mmHg) | 1.00 | 0.99-1.01 | 0.150 |
| Anion gap (mmol/L) | 1.00 | 0.97-1.02 | 0.963 |
| Bicarbonate (mmol/L) | 1.01 | 0.98-1.03 | 0.522 |
| Lactate (mmol/L) | 1.02 | 0.96-1.08 | 0.492 |
| Heart rate (beats/minute) | 1.00 | 0.99-1.01 | 0.974 |
| Respiratory rate (beats/minute) | 0.99 | 0.97-1.01 | 0.571 |
| Temperature (°C) | 0.91 | 0.81-1.01 | 0.103 |
| MBP (mmHg) | 0.99 | 0.98-0.99 | 0.007 |
| SPO_2_ (%) | 0.99 | 0.96-1.02 | 0.709 |
| COPD | 1.66 | 1.09-2.52 | 0.016 |
| Congestive heart failure | 1.91 | 1.24-2.95 | 0.003 |
| Diabetes | 1.61 | 1.24-2.10 | < 0.001 |
| Hypertension | 0.89 | 0.68-1.16 | 0.402 |
| Charlson comorbidity index | 1.20 | 1.15-1.25 | < 0.001 |
| SAPS II | 1.02 | 1.01-1.03 | < 0.001 |
| SOFA | 1.06 | 1.03-1.09 | < 0.001 |
| Sepsis | 1.52 | 1.17-1.98 | 0.002 |
| CRRT | 1.37 | 1.02-1.84 | 0.033 |
| Ventilation | 0.87 | 0.46-1.65 | 0.683 |
| Vasoactive agent | 1.33 | 0.94-1.87 | 0.104 |

**Supplemental Table 4: Multivariable Cox Regression Analysis of 90-day Mortality Among CCI Patients** **Who Survived Post-Discharge.**

|  | **Model I** | | | **Model II** | | **Model III** | |
| --- | --- | --- | --- | --- | --- | --- | --- |
| **Varables** | | **HR（95%CI）** | **p value** | **HR（95%CI）** | **p value** | **HR（95%CI）** | **p value** |
| **BMI** | | 0.97 (0.95-0.99) | 0.010 | 0.98 (0.96-0.99) | 0.034 | 0.97 (0.95-0.99) | 0.008 |
| **Under-weight** | | 0.70 (0.30-1.62) | 0.415 | 0.63 (0.27-1.46) | 0.291 | 0.62 (0.26-1.46) | 0.284 |
| **Normal** | | reference | reference | reference | reference | reference | reference |
| **Overweight** | | 0.67 (0.48-0.93) | 0.017 | 0.68 (0.49-0.95) | 0.013 | 0.61 (0.43-0.83) | < 0.001 |
| **Obesity class I group**  **Obesity class II group**  **Obesity class III group** | | 0.53 (0.35-0.79)  0.53 (0.32-0.88)  0.64 (0.40-0.12) | 0.002  0.044  0.062 | 0.56 (0.37-0.84)  0.52 (0.31-0.86)  0.71 (0.44-1.14) | 0.005  0.011  0.160 | 0.48 (0.32-0.72)  0.43 (0.26-0.72)  0.58 (0.35-0.94) | < 0.001  0.001  0.001 |

Model I adjusted for none;

Model II adjusted for age, gender, race and admission types;

Model III adjusted for 41 potential variables included demographic characteristics, laboratory parameters, blood gas, vital signs, comorbidities, severity scoring systems, organ support and diagnosis by stepwise Cox regression. The result shows that age, BMI, admission type, PaCO2, potassium, sodium, charlson comorbidity index, SOFA score and sepsis were influence factor of 90-day mortality in patients with CCI who survived post-discharge.

**Supplement Figure 3: The association between BMI and 90-day mortality in CCI patients who survived past discharge, adjusted for confounding factors. Dashed vertical lines represent WHO BMI category thresholds of 18.5 kg/m^2 (underweight to healthy), 25 kg/m^2 (healthy weight to overweight), and 30 kg/m^2 (overweight to obese).**


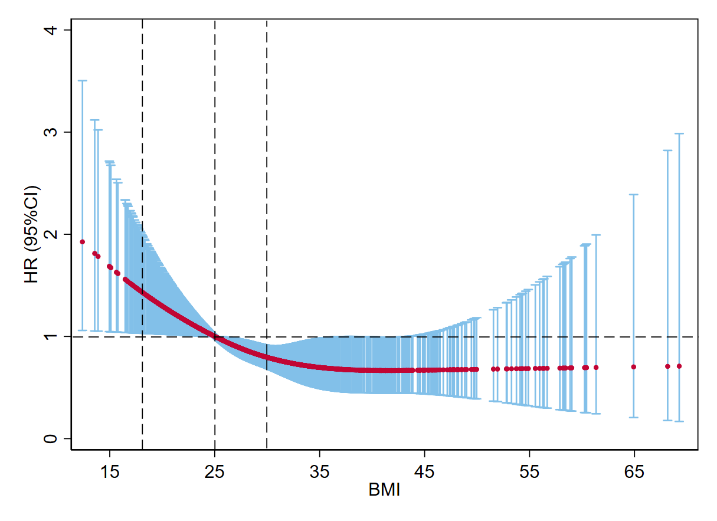

Supplement: Supplementary file 1 — Supplementary tables and figures. [file 40001_2024_1962_MOESM1_ESM.docx]
